# Prevalence of CADASIL and Fabry Disease in a Cohort of MRI Defined Younger Onset Lacunar Stroke

**DOI:** 10.1371/journal.pone.0136352

**Published:** 2015-08-25

**Authors:** Laura L. Kilarski, Loes C. A. Rutten-Jacobs, Steve Bevan, Rob Baker, Ahamad Hassan, Derralynn A. Hughes, Hugh S. Markus, UK Young Lacunar Stroke DNA Study

**Affiliations:** 1 Stroke and Dementia Research Centre, St George’s University of London, London, United Kingdom; 2 Department of Clinical Neurosciences, University of Cambridge, Cambridge, United Kingdom; 3 Department of Haematology, Lysosomal Storage Disorders Unit, Royal Free Hospital and University College Medical School, London, United Kingdom; 4 Department of neurology, Leeds General Infirmary, Leeds, United Kingdom; King Faisal Specialist Hospital and Research center, SAUDI ARABIA

## Abstract

**Background and Purpose:**

Cerebral autosomal dominant arteriopathy with subcortical infarcts and leukoencephalopathy (CADASIL), caused by mutations in the *NOTCH3* gene, is the most common monogenic disorder causing lacunar stroke and cerebral small vessel disease (SVD). Fabry disease (FD) due to mutations in the *GLA* gene has been suggested as an underdiagnosed cause of stroke, and one feature is SVD. Previous studies reported varying prevalence of CADASIL and FD in stroke, likely due to varying subtypes studied; no studies have looked at a large cohort of younger onset SVD. We determined the prevalence in a well-defined, MRI-verified cohort of apparently sporadic patients with lacunar infarct.

**Methods:**

Caucasian patients with lacunar infarction, aged ≤70 years (mean age 56.7 (SD8.6)), were recruited from 72 specialist stroke centres throughout the UK as part of the Young Lacunar Stroke DNA Resource. Patients with a previously confirmed monogenic cause of stroke were excluded. All MRI’s and clinical histories were reviewed centrally. Screening was performed for *NOTCH3* and *GLA* mutations.

**Results:**

Of 994 subjects five had pathogenic *NOTCH3* mutations (R169C, R207C, R587C, C1222G and C323S) all resulting in loss or gain of a cysteine in the *NOTCH3* protein. All five patients had confluent leukoaraiosis (Fazekas grade ≥2). CADASIL prevalence overall was 0.5% (95% CI 0.2%-1.1%) and among cases with confluent leukoaraiosis 1.5% (95% CI 0.6%-3.3%). No classic pathogenic FD mutations were found; one patient had a missense mutation (R118C), associated with late-onset FD.

**Conclusion:**

CADASIL cases are rare and only detected in SVD patients with confluent leukoaraiosis. No definite FD cases were detected.

## Background

Lacunar infarction, resulting from occlusion of the cerebral small vessels, accounts for a quarter of all ischemic strokes. Family history studies support a role for genetic factors in the risk of lacunar stroke [1, 2], and a number of monogenic cause of cerebral small vessel disease (SVD) have been described. The most common of these is cerebral autosomal dominant arteriopathy with subcortical infarcts and leukoencephalopathy (CADASIL), caused by mutations in the *NOTCH3* gene.

The main clinical symptoms of CADASIL are migraine with aura, lacunar strokes, progressive cognitive deficits and depression [3]. Another monogenic disorder that causes SVD is Fabry disease (FD), an X-linked lysosomal storage disorder, caused by mutations in the *GLA* gene. The resulting alpha-galactosidase A deficiency causes accumulation of glycolipids in the walls of small blood vessels, nerves, glomerular and tubular epithelial cells, and cardiomyocytes [4]. Both lacunar stroke and radiological appearances of SVD on MRI have been described as a feature of the disease [5].

It has been suggested both FD and CADASIL are underdiagnosed [6, 7]. Previous studies reported a varying prevalence of CADASIL and FD in stroke [8–11], most likely due to varying methods and phenotypes studied. Few studies have looked at the prevalence of these diseases in large cohorts with apparently sporadic SVD. In addition, no studies have looked at a large MRI-defined cohort of younger onset lacunar stroke.

We determined the prevalence of pathogenic *NOTCH3* and *GLA* mutations in a large well-defined, MRI verified cohort of patients with apparently sporadic lacunar stroke.

## Methods

### Study population

This study is a part of the Young Lacunar Stroke DNA study, a multicentre cohort study, which constitutes a large DNA resource of young patients with well phenotyped lacunar stroke and stroke-free community controls.

Between 2005 and 2012, 1247 patients with suspected lacunar stroke without a known monogenic cause were recruited from 72 specialist stroke centres throughout the UK (S1 Table). Collection was as part of a single study with an identical study protocol, and identical inclusion and exclusion criteria as well as a single data collection form used in all participating centres. Inclusion criteria were age of stroke < 70 years, MRI confirmed lacunar stroke and European ancestry.

Lacunar stroke was defined as a clinical lacunar syndrome [12], with a compatible lesion on MRI (subcortical infarct ≤15 mm in diameter). Patients had to have had a clinical MRI for inclusion, and imaging of the carotid arteries and ECG was mandatory. Echocardiography was performed when appropriate. All MRI’s and clinical histories were reviewed centrally in a single co-ordinating centre by one physician (HM). Exclusion criteria were: stenosis > 50% in the extra- or intracranial cerebral vessels, or previous carotid endarterectomy; cardioembolic source of stroke, defined according to the TOAST (Trial of Org 10172 in Acute Stroke Treatment) criteria [13], as high or moderate probability; cortical infarct on MRI; subcortical infarct > 15mm in diameter, as these can be caused by embolic mechanisms (striatocapsular infarcts); any other specific cause of stroke (e.g. lupus anticoagulant, cerebral vasculitis, dissection) and known monogenic forms of stroke e.g. CADASIL). 1030 patients met the inclusion criteria of the main study and 994 patients had DNA of sufficient quality available in which screening for CADASIL and FD was performed. The study was approved by the Multi-Centre Research Ethics Committee for Scotland (04/MRE00/36) and written informed consent was obtained from all participants. Leukoaraiosis was graded on MRI using the semi-quantitative Fazekas scale which has been shown to reflect pathological severity of SVD in a post-mortem validation study [14]. All MRI’s and clinical histories were reviewed centrally by one physician (HM). Patients were considered to have leukoaraiosis if they had a Fazekas grade ≧2. In addition in patients found to be CADASIL positive white matter hyperintensities in the anterior temporal pole and the external capsule, characteristic locations for CADASIL, where graded using the Scheltens scale [15]. Central review of all cases including MRIs was performed blinded to the results of the genetic analysis.

### Cardiovascular risk factors

Hypertension was defined as taking antihypertensive treatment or blood pressure greater than 160 mmHg systolic or 90mmHg or diastolic one week or more after acute stroke onset. Hypercholesterolemia was defined as on drug treatment of cholesterol > 5.2mmol/l.

### Genetic analysis

DNA was extracted and purified from whole blood (collected in EDTA) using the Nucleon BACC3 genomic DNA extraction kit (Tepnel Life Sciences, Manchester, UK) or the Chemagic Blood 3k DNA kit in conjunction with a Magnetic Separation Module 1 (PerkinElmer Chemagen Technologie GmbH, Baesweiler).

The *NOTCH3* gene was screened by denaturing high performance liquid chromatography (DHPLC) using the WAVE 3500HT system (Transgenomic Ltd., Glasgow, UK). Exons 3, 4 5, 6, 8, 11, 18, 19 and 22 were screened. This screen has been shown to detect 90% of mutations in a UK population [16]. This was followed by Sanger sequencing of exons 3 and 4 in all cases and sequencing of samples with a suspected variant in any other exon. Fragments were amplified using polymerase chain reaction (PCR), cleaned up using Exonuclease and FastAP Thermosensitive Alkaline Phosphatase (Thermo Scientific, UK), and sequenced using a BigDye Terminator Cycle Sequencing kit (Perkin Elmer Applied Biosystems, Cheshire, UK) and analysed on an ABI3100 sequencer (Applied Biosystems, Foster City, CA) according to the manufacturer's instructions. Sequences were analysed using Sequencher 5.0 (Gene Codes, Ann Arbor, MI, USA).

The *GLA* gene was screened using high resolution melt-curve analysis on an ABI7500 Fast Real-Time PCR platform, using ABI HRM Mastermix in 9 separate PCR reactions, covering all exons, all intron/exon junctions and one specific reaction aimed at a common deep intronic mutation. Primer sequences are found in a paper by Tai and colleagues.[17] Samples with a suspected variant were sequenced on an ABI3500XL automated sequencer using ABI BigDye v1.1. Prior to setting up the sequencing reaction, PCR products were cleaned up using ExoSap-IT (Affymetrix, High Wycombe, UK) and excess dye terminators were removed using the DyeEx 96 system (Qiagen, Manchester, UK). Sequences were analysed using Mutation Surveyor software (SoftGenetics, State College, PA, USA).

Nonsynonymous variants were evaluated for pathogenicity by *in silico* mutation prediction tools, using three algorithms (PolyPhen-2 [18], SIFT [19] and Mutation Taster [20]).

## Results

Patient characteristics are shown in Table 1. There were 617 patients (62.1%) with first stroke onset at ≤60 years. A history of stroke in first-degree relatives was reported in 427 patients (43.0%).

**Table 1:** Patient characteristics.

Age, mean years (SD)	56.7 (8.6)
Male, n (%)	706 (71.0)
Hypertension, n (%)	714 (71.8)
Diabetes, n (%)	165 (16.6)
Hypercholesterolemia, n (%)	671 (67.7)
Ever smoker, n (%)	698 (70.2)
Alcohol ≥ 20 u/week	290 (29.2)
BMI, mean kg/m^2^ (SD)	28.7 (6.2)
Migraine, n (%)	195 (19.6)
MI, n (%)	35 (3.5)
PVD, n (%)	28 (2.8)
Recurrent strokes, n (%)	77 (7.7)
Leukoaraiosis (Fazekas grade ≥2), n (%)	329 (33.1)
Number of lacunes, median (IQR)	2 (1–2)
Microbleeds present^a^, n (%)	63 (18.6)

Abbreviations: SD: standard deviation; IQR: interquartile range.

^a^ Gradient echo imaging was available in 339 (34%) patients.

**Fig 1:**
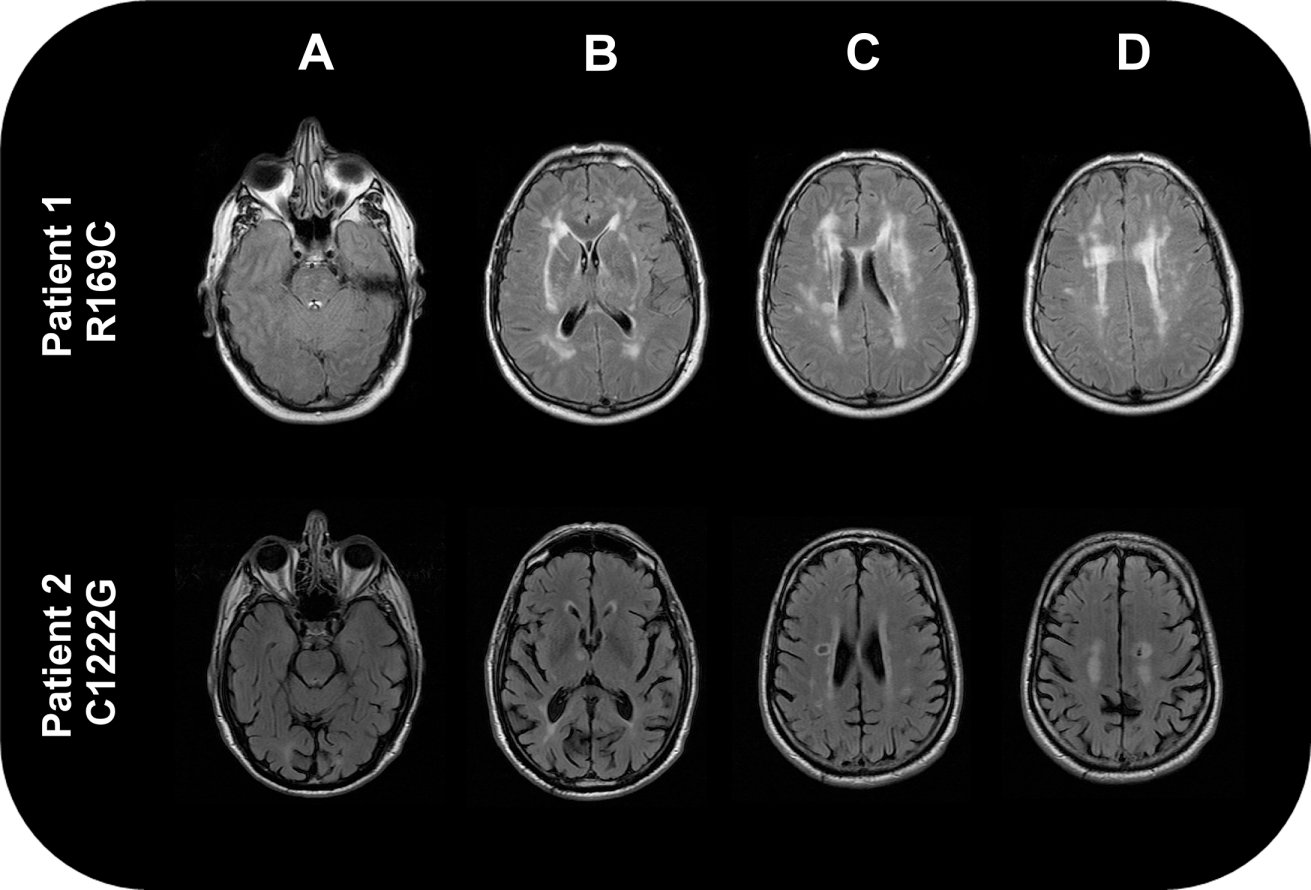
FLAIR Brain MRIs scans from two of the patients with notch 3 mutations. The upper panel 1 shows images from patient 1 (R169C), and the lower panel two shows images from patient 2 panel 2 (C1222G). These show leukoaraiosis in both cases (images C and D) but minimal anterior pole involvement in patient 1 (image upper panel A) and no involvement in patient 2 (image lower panel A). Patient 1 also shows confluent external capsule involvement (image B), but this is absent from patient 4 (lower panel B).

Five patients had pathogenic *NOTCH3* mutations (c.505C>T, R169C; c.619C>T, R207C; c.1759C>T, R587C; c.3664T>G, C1222G; c.967T>A, C323S) all resulting in loss or gain of a cysteine in the *NOTCH3* protein. Clinical characteristics of these five patients are reported in Table 2. All five cases had confluent leukoaraiosis, but there were few non-stroke clinical features of CADASIL (Fig 1). Only two had migraine and both of these were migraine without aura. Three cases had no family history of stroke. On MRI review three cases had no anterior temporal pole involvement, while it was mild (Schelten’s grades 1 and 2) in the other two cases.

**Table 2:** Characteristics of patients with a *NOTCH3* mutation.

	Patient 1	Patient 2	Patient 3	Patient 4	Patient 5
**Mutation**	R169C	C1222G	R207C	R587C	C323S
**Demographics and vascular risk factors**					
Age at first stroke (years)	45	55	58	65	34
Sex	Male	Male	Male	Female	Male
Hypertension	No	Yes	Yes	Yes	No
Diabetes	No	Yes	No	No	No
Hyperlipidaemia	Yes	Yes	Yes	Yes	Yes
Ever smoker	Yes	Yes	Yes	Yes	Yes
Alcohol ≥ 20 units/week	No	No	No	No	Yes
Body mass index (kg/m^2^)	30	26	25	27	22
Depression	No	No	No	No	No
Migraine	Yes, no aura	No	Yes, no aura	No	No
Myocardial infarction	No	No	No	No	No
Peripheral vascular disease	No	No	No	No	No
Recurrent strokes	No	Yes	Yes	No	No
Family history of stroke	Father, age 36	No	Mother, age 70	No	No
**Brain MRI characteristics**					
Leukoaraiosis (Fazekas grade, 1–3)	3	2	3	3	2
Number of lacunes	3	3	2	1	2
Microbleeds	NA	No	NA	No	No
Anterior temporal pole WMH (Scheltens scale, 1–6)	1	0	0	0	2
External capsule WMH (Scheltens scale, 1–6)	5	0	3	6	0

Abbreviations: WMH, white matter hyperintensities; NA, not available.

The overall mutation carrier frequency was 0.5% (95% CI 0.2%-1.1%), while among cases with confluent leukoaraiosis it was 1.5% (95% CI 0.6%-3.3%). Comparing age groups, the overall mutation carrier frequency was 0.6% (95% CI 0.2%-1.6%) in patients aged ≤ 60 years and 0.3% (95% CI 0.01%-1.3%) in patients aged >60 years. Among cases with confluent leukoaraiosis the mutation carrier frequency was 1.9% (95% CI 0.5%-5.0%) in patients aged ≤ 60 years and 0.6% (95% CI 0.03%-2.9%) in patients aged >60 years.

In addition to the reported pathogenic mutations, two novel *NOTCH3* missense variants (c.319C>T, R107W and c.3552C>G, D1184E) were identified that do not disrupt the number of cysteine residues in any EGF-like domains. However, both novel missense mutations were predicted by Polyphen2 as probably damaging, SIFT predicted only R107W to affect protein function and MutationTaster predicted only D1184E to be pathogenic. Clinical characteristics of these two patients are reported in Table 3.

**Table 3:** Clinical characteristics of cases with genetic variants of unknown significance.

	Patient 5	Patient 6	Patient 7
Gene	*NOTCH3*	*NOTCH3*	*GLA*
Mutation	R107W	D1184E	R118C
Age at event (years)	63	58	56
Sex	Male	Female	Male
Hypertension	Yes	Yes	Yes
Diabetes	No	Yes	Yes
Hyperlipidaemia	No	Yes	Yes
Ever smoker	Yes	Yes	Yes
Alcohol ≥ 20 u/week	No	No	No
Body mass index, (kg/m^2^)	32	36	27
Depression	No	No	No
Migraine	No	No	No
Myocardial infarction	No	No	Yes
Peripheral vascular disease	No	No	No
Renal function	Normal	Normal	Normal
Recurrent strokes	No	No	Yes
Family history of stroke	Mother, age unknown	Two siblings, ages 47 & 56	Mother, age 61
Leukoaraiosis (Fazekas grade ≥2)	No	Yes	No

**Fig 2:**
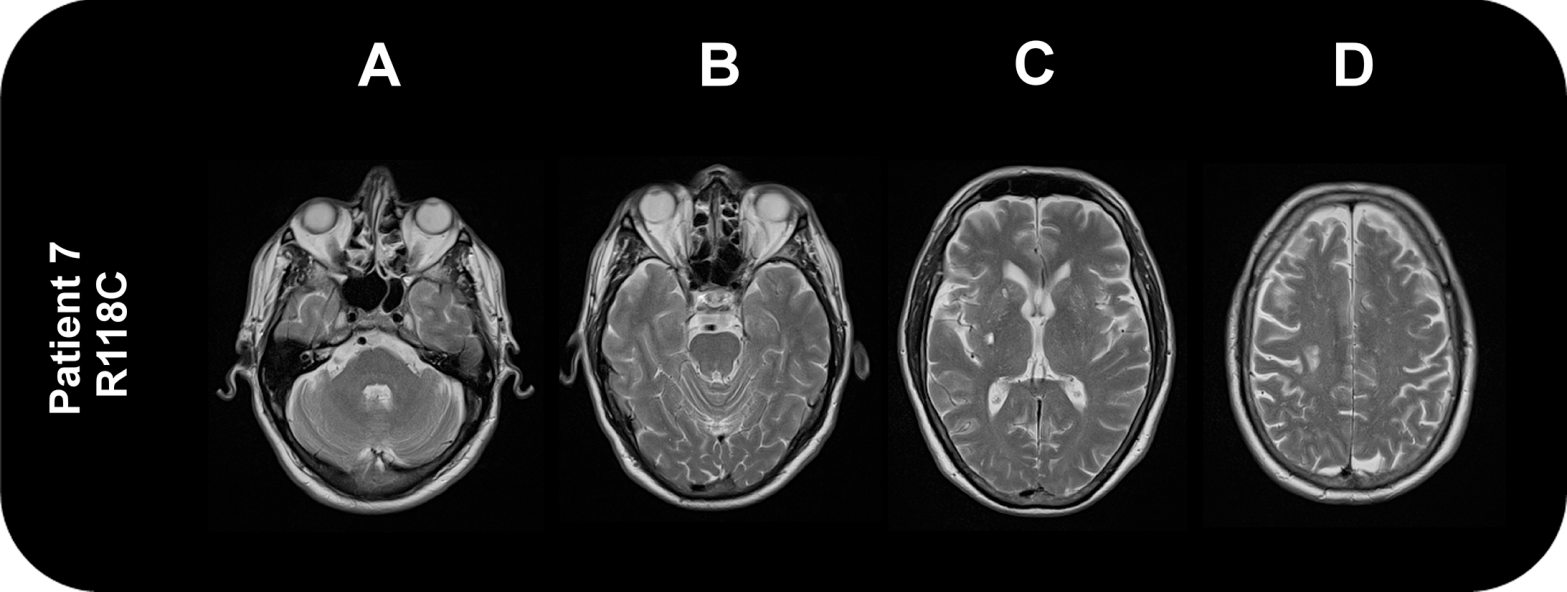
Brain MRI (T2-Weighted) of the patient carrying the *GLA* mutation R118C. Two lacunes are present in the right caudate nucleus and external capsule (C). An area of white matter hyperintensity is present in the peripheral right posterior frontal white matter (D). There were no abnormalities in the posterior circulation.

None of the patients had a nonsense mutation in the *GLA* gene known to cause classical FD. One missense mutation (c.352C>T, R118C) was identified, which has been suggested to be a mild or late-onset variant [7, 21]. Clinical characteristics of this patient are reported in Table 3 and Fig 2 shows the corresponding brain MRI.

## Discussion

We present the results of a comprehensive screening study of the *NOTCH3* gene for CADASIL and the *GLA* gene for FD in a large cohort of MRI confirmed young lacunar stroke. A few cases of CADASIL were detected giving an overall prevalence of 0.5%, although this rose to 1.5% when only patients with confluent leukoaraiosis were considered. We found only one case of a *GLA* mutation possibly associated with Fabry disease.

Only a few studies have investigated the prevalence of CADASIL in unselected ischemic stroke patients [8, 9, 11]. The reported prevalence in these studies varied between 0% and 4%, which is probably due to the phenotypic variation and small numbers. In the current study we reduced the phenotypic variation by investigating a large sample of patients with MRI-defined lacunar stroke, which is the typical stroke subtype in CADASIL patients.

Our findings are in line with the mutation frequency reported in a much smaller previous study.[9] The CADASIL mutations were typical point mutations resulting in a loss or gain of a cysteine residue in an EGF-like repeat. Two were novel (C1222G and C323S) while R169C and R207C have been previously described in Caucasian CADASIL patients [22, 23], whereas the R587C mutation only has been reported in a Korean CADASIL patient [24]. The cases identified in the current study had few non-stroke clinical or MRI features of CADASIL. An explanation for this is that any cases with obvious characteristics of CADASIL would have been excluded, as an exclusion criteria was a diagnosis of CADASIL at presentation. Furthermore, traditional cardiovascular risk factors were common in these patients, which might have led to lack of suspicion.

In addition, two novel variants were found (R107W and D1184E), also located in one of the EGF-like repeats, but not involving any cysteine residues. Although *in silico* mutation prediction tools predicted these mutations to be probably pathogenic, functional studies are needed to confirm this pathogenicity. Other non-cysteine affecting *NOTCH3* mutations have been described in SVD, although their pathogenicity has been debated [25–27].

A potential limitation of the current study is that not all of the exons encoding the extracellular portion of the Notch 3 protein in which CADASIL mutations occur were screened. However, it has been shown that mutations in the *NOTCH3* exons screened in this study (3–6, 8, 11, 18, 19, and 22) account for 90% of CADASIL mutations in the UK population in which this study was performed [16, 22, 28].

A study in young-onset cryptogenic stroke patients reported a prevalence of FD as high as 4.9% in males and 2.4% in females [29], although subsequent studies in young onset stroke patients have found lower prevalence (0%-2.4% in cryptogenic stroke and 0%-1% in all ischemic stroke) [21, 30–36]. Although FD is associated with a number of stroke mechanisms including cardioembolism, and disease of the vertebrobasilar arteries, small vessel disease is a recognized feature and white matter hyperintensities on MRI are common [5]. No previous studies have investigated the prevalence of FD in large cohorts of MRI defined lacunar stroke. We found only one missense mutation in the *GLA* gene (R118C); it is unclear if this was related to FD but an association late onset FD has been previously suggested [7, 21]. In contrast to mutations causing classical FD, R118C is associated with relatively high residual α-galactosidase-A activity, lack of accumulation of lyso-Gb3 and carriers exhibit few symptoms that might be suggestive of FD [7, 21, 37]. Because testing was performed on stored blood samples on an anonymous basis, we were not able to carry further tests on the patient to confirm the diagnosis. Despite FD being an X-linked disease, cases of stroke due to *GLA* mutations have been described in women as well as men. However, in women accurate diagnosis cannot be made by blood activity of alpha-galactosidase A, and therefore we performed genotyping for causative mutations in both genders. Although the yield of FD screening seems to be low in lacunar stroke patients, the yield of FD screening may be higher in other stroke populations. Small vessel disease is only one of the stroke subtypes associated with Fabry disease and other presentation include cardioembolism and otherwise cryptogenic stroke. It is possible that the yield of Fabry screening may be higher in other groups of young stroke patients including patients with cryptogenic stroke, or in patients who have signs of systemic disease such as renal disease.

In conclusion we aimed to provide an estimate of the prevalence of mutations causing CADASIL and FD in a large British cohort of patients presenting with apparently sporadic lacunar stroke. Our study suggests that CADASIL is only present in patients with leukoaraiosis, in whom the prevalence was 1.5%. The CADASIL patients that were detected in the present study had conventional risk factors and not a MRI scan typical for CADASIL, which might have led to lack of suspicion at presentation. Therefore, the presence of conventional risk factors and the absence of typical CADASIL characteristics on MRI should not necessarily preclude screening in younger onset lacunar stroke patients, particularly if there is confluent leukoaraiosis. Screening for FD has a very low pick up in this patient group.

## Supporting Information

S1 Table List of participating centres, local investigators and number of included patients per centre. (DOCX)
