# Supplementary material for: Prevalence of CADASIL and Fabry Disease in a Cohort of MRI Defined Younger Onset Lacunar Stroke
Source: PLoS One. 2015 Aug 25;10(8):e0136352. doi: 10.1371/journal.pone.0136352 (PMC4549151; doi:10.1371/journal.pone.0136352)
Supplement: S1 Table — (DOCX) [file pone.0136352.s001.docx]

**S1 Supplemental Information**

**Participating centres**

| **Centre** | ***n***  **patients** | **Local investigators** |
| --- | --- | --- |
| Aberdeen Royal Infirmary, Aberdeen | 12 | Mary Macleod |
| Addenbrooke’s Hospital, Cambridge | 54 | Jean-Claude Baron, Elizabeth Warburton, Diana J Day, Julie White |
| Airedale General Hospital, Steeton | 4 | Samantha Mawer |
| Barnsley Hospital, Barnsley | 3 | Mohammad Albazzaz, Pravin Torane, Keith Elliott, Kay Hawley |
| Bart’s and the London, London | 2 | Patrick Gompertz |
| Basingstoke and North Hampshire Hospital, Basingstoke | 13 | Elio Giallombardo, Deborah Dellafera |
| Blackpool Victoria Hospital, Blackpool | 11 | Mark O'Donnell |
| Bradford Royal Infirmary, Bradford | 1 | Chris Patterson |
| Bristol Royal Infirmary, Bristol | 8 | Sarah Caine |
| Charing Cross Hospital, London | 12 | Pankaj Sharma |
| Cheltenham General and Gloucester Royal Hospitals, Cheltenham and Gloucester | 10 | Dipankar Dutta |
| Chesterfield Royal Hospital, Chesterfield | 4 | Sunil Punnoose, Mahmud Sajid |
| Countess of Chester Hospital, Chester | 22 | Kausik Chatterjee |
| Derriford Hospital, Plymouth | 4 | Azlisham Mohd Nor |
| Dorset County Hospital NHS Foundation Trust, Dorchester | 6 | Rob Williams |
| East Kent Hospitals University NHS Foundation Trust, Kent | 22 | Hardeep Baht, Guna Gunathilagan |
| Eastbourne District General Hospital, Eastbourne | 4 | Conrad Athulathmudali |
| Frenchay Hospital, Bristol | 1 | Neil Baldwin |
| Frimley Park Hospital NHS Foundation Trust, Frimley | 6 | Brian Clarke |
| Guy’s and St Thomas’ Hospital, London | 14 | Tony Rudd |
| Institute of Neurology, London | 25 | Martin Brown |
| James Paget University Hospital, Great Yarmouth | 1 | Peter Harrison |
| King's College Hospital, London | 16 | Lalit Kalra |
| Leeds Teaching Hospitals NHS Trust, London | 126 | Ahamad Hassan |
| Leicester General Hospital and Royal Infirmary, Leicester | 9 | Tom Robinson, Amit Mistri |
| Luton and Dunstable NHSFT University Hospital, Luton | 16 | Lakshmanan Sekaran, Sakthivel Sethuraman, Frances Justin |
| Maidstone andTunbridge Wells NHS Trust | 3 | Peter Maskell |
| Mayday University Hospital, Croydon | 14 | Enas Lawrence |
| Medway Maritime Hospital, Gillingham | 5 | Sam Sanmuganathan |
| Milton Keynes Hospital, Milton Keynes | 1 | Yaw Duodu |
| Musgrove Park Hospital, Taunton | 9 | Malik Hussain |
| Newcastle Hospitals NHS Foundation Trust, Newcastle upon Tyne | 12 | Gary Ford |
| Ninewells Hospital, Dundee | 5 | Ronald MacWalter |
| North Devon District Hospital, Barnstaple | 8 | Mervyn Dent |
| Nottingham University Hospitals, Nottingham | 17 | Philip Bath, Fiona Hammonds |
| Perth Royal Infirmary, Perth | 2 | Stuart Johnston |
| Peterborough City Hospital, Peterborough | 1 | Peter Owusu-Agyei |
| Queen Elizabeth Hospital, Gateshead | 5 | Tim Cassidy, Maria Bokhari |
| Radcliffe Infirmary, Oxford | 5 | Peter Rothwell |
| Rochdale Infirmary, Rochdale | 4 | Robert Namushi |
| Rotherham General Hospital, Rotherham | 1 | James Okwera |
| Royal Cornwall Hospitals NHS Trust, Truro | 11 | Frances Harrington, Gillian Courtauld |
| Royal Devon and Exeter Hospital, Exeter | 22 | Martin James |
| Royal Hallamshire Hospital, Sheffield | 1 | Graham Venables |
| Royal Liverpool University Hospital and Broadgreen Hospital, Liverpool | 9 | Aravind Manoj |
| Royal Preston Hospital, Preston | 18 | Shuja Punekar |
| Royal Surrey County Hospital, Guildford | 23 | Adrian Blight, Kath Pasco |
| Royal Sussex County Hospital, Brighton | 14 | Chakravarthi Rajkumar, Joanna Breeds |
| Royal United Hospital, Bath | 6 | Louise Shaw, Barbara Madigan |
| Salford Royal Hospital, Salford | 16 | Jane Molloy |
| Southampton General Hospital, Southampton | 1 | Giles Durward |
| Southend Hospital, Westcliff-on-Sea | 26 | Paul Guyler |
| Southern General Hospital, Glasgow | 34 | Keith Muir, Wilma Smith |
| St George’s Hospital, London | 108 | Hugh Markus |
| St Helier Hospital, Carshalton | 10 | Val Jones |
| Stepping Hill Hospital, Stockport | 4 | Shivakumar Krishnamoorthy |
| Sunderland Royal Hospital, Sunderland | 1 | Nikhil Majumdar |
| The Royal Bournemouth Hospital, Bournemouth | 15 | Damian Jenkinson |
| The Walton Centre, Liverpool | 15 | Richard White |
| Torbay Hospital, Torquay | 19 | Debs Kelly |
| University Hospital Aintree, Liverpool | 19 | Ramesh Durairaj |
| University Hospital of North Staffordshire, Stoke-on-trent | 16 | David Wilcock |
| Wansbeck General Hospital and North Tyneside Hospital, Ashington and North Shields | 6 | Christopher Price |
| West Cumberland Hospital, Whitehaven | 6 | Olu Orugun, Rachel Glover |
| West Hertfordshire Hospital, Watford | 20 | David Collas |
| Western General Hospital, Edinburgh | 12 | Cathie Sudlow |
| Western Infirmary, Glasgow | 33 | Kennedy R. Lees, Jesse Dawson |
| Wycombe Hospital and Stoke Mandeville, High Wycombe | 20 | Dennis Briley and Matthew Burn |
| Yeovil District Hospital, Yeovil | 46 | Khalid Rashed |
| York Teaching Hospital, York | 1 | John Coyle |
